# Disruption of Selenium Handling During Puberty Causes Sex-Specific Neurological Impairments in Mice

**DOI:** 10.3390/antiox8040110

**Published:** 2019-04-24

**Authors:** Penny M. Kremer, Daniel J. Torres, Ann C. Hashimoto, Marla J. Berry

**Affiliations:** Department of Cell and Molecular Biology, John A. Burns School of Medicine, University of Hawaii, Honolulu, HI 96813, USA; pmkremer@hawaii.edu (P.M.K.); djtorr@hawaii.edu (D.J.T.); ahashimo@hawaii.edu (A.C.H.)

**Keywords:** selenium, selenoprotein, neurodegeneration, neurodevelopment, sex differences

## Abstract

Selenium is an essential trace element linked to normal development and antioxidant defense mechanisms through its incorporation into selenoproteins via the amino acid, selenocysteine (Sec). Male mice lacking both the Se transporter, selenoprotein P (SELENOP), and selenocysteine lyase (Scly), which plays a role in intracellular Se utilization, require Se supplementation for viability and exhibit neuromotor deficits. Previously, we demonstrated that male SELENOP/Scly double knockout (DKO) mice suffer from loss of motor function and audiogenic seizures due to neurodegeneration, both of which are alleviated by prepubescent castration. The current study examined the neuromotor function of female DKO mice using the rotarod and open field test, as well as the effects of dietary Se restriction. Female DKO mice exhibited a milder form of neurological impairment than their male counterparts. This impairment is exacerbated by removal of Se supplementation during puberty. These results indicate there is a critical time frame in which Se supplementation is essential for neurodevelopment. These sex-specific differences may unveil new insights into dietary requirements for this essential nutrient in humans.

## 1. Introduction

Selenium is an essential micronutrient due to its presence at the catalytic center of selenoenzymes, many of which have been implicated in biological functions that are crucial for human health [1]. Selenium is acquired through diet and incorporated into selenoproteins as the amino acid, selenocysteine (Sec). Previous studies have shown that Se is preferentially maintained in both brain and testes under Se-compromised conditions, although the brain seems to have priority over the testes [2]. Selenium is critical for proper brain function and may play a significant protective role against neurodegenerative diseases [3]. Dietary Se is absorbed in the gut, and enters the liver via the portal vein, wherein it is incorporated into multiple Sec residues in the Se-transporter, Selenoprotein P (SELENOP, formerly SelP or Sepp1). Selenoprotein P is secreted from the liver into the plasma, transported to peripheral tissues and taken up in neurons in the brain and Sertoli cells in the testes through apolipoprotein E2 receptor (ApoER2)-mediated endocytosis [2]. Selenocysteine lyase (Scly), expressed in most tissues, but at highest levels in liver, kidney, and testes, followed by the brain, heart, and spleen [4], catalyzes the breakdown of Sec, allowing Se to be recycled for additional selenoprotein synthesis [5]. Disruption of Se transport or recycling results in tissue-specific alterations in Se content and selenoprotein expression, accompanied by sex-specific phenotypic consequences. Previous studies showed that SELENOP knockout (KO) mice exhibit neurological motor deficits, including ataxia, loss of motor coordination, and wide clumsy gait [6], that predominate in males [7]. Loss of Scly results in sex-specific imbalances in energy metabolism in mice, accompanied by mild neurological effects [7,8].

Previously, we bred SELENOP KO and Scly KO strains to generate double knockout (DKO) mice and determined that Se supplementation was required for viability of male DKO (MDKO) pups [9]. Thus, all DKO dams and pups were maintained with supplemental Se in drinking water (0.78 ppm Se) and are designated DKOSe. This level of Se supplementation in drinking water is approximately three times higher than that present in Se-adequate chow. Even with Se supplementation, MDKOSe mice exhibited decreased survival and severe neurological impairment, whereas females exhibited only mild neurological deficits [9]. Strikingly, we found that prepubescent castration attenuated this neurodegeneration [10].

Based on the more pronounced effects of SELENOP KO, Scly KO, and DKOSe in male mice, our initial studies focused primarily on males, with limited numbers of females included for comparison. Herein, we have extended our studies to undertake a more extensive characterization of female DKO mice maintained on supplemental Se (FDKOSe), comparing them with MDKOSe mice, and both male and female wild-type (WTSe) mice maintained on the same regimen. We also investigated the effects of removing supplemental Se at varying ages. 

The results reported herein show that Se supplementation is crucial in early life for DKO mice of both sexes, and both exhibit neurological deficits even with Se supplementation, but the severity of the phenotype is vastly different in males versus females. We previously reported a decline in male viability beginning around puberty, whereas females are fully viable [8]. Removal of Se supplementation at this stage exacerbates the neurological phenotype in females, but would likely accelerate lethality in males. These sex-specific distinctions in Se requirements and utilization underlie the critical need for careful evaluation of sex as a biological variable in nutrient and other dietary requirements, as well as in many other aspects of biology and health. 

## 2. Materials and Methods

### 2.1. Animals

Male and female C57/BL6N WT and DKOSe mice were generated from breeders in our colony as previously described [9]. Because DKOSe mice require Se supplementation for survival, all dams for DKO and WT controls received ad libitum access to standard chow (~0.25 ppm Se) and water supplemented with 10 µM sodium selenite, Na_2_SeO_3_, (~0.78 ppm Se), hereafter SeH_2_O. Pups remained on SeH_2_O after weaning either until sacrifice or as indicated. Animals were weighed and sacrificed on day 70 (P70) and tissue collected thereafter. Brains were either collected and weighed following perfusion with 4% paraformaldehyde (PFA) for histological analysis or dissected and snap frozen in liquid nitrogen following CO_2_ euthanasia and stored at −80 °C until use for enzymatic activity analysis of specific brain regions. All procedures and experimental protocols involving animals were approved by the University of Hawaii’s Institutional Animal Care and Use Committee. Animal Care and Use Committee (IACUC) Protocol: “Mechanism of Selenoprotein Synthesis and Studies of Selenoprotein Functions”: APN 09-871-9, approved: 16 August 2018. Institutional Biosafety Committee (IBC) Protocol: “Mechanism of Selenoprotein Synthesis and Studies of Selenoprotein Functions”: IBC #18-10-544-02-4A-1R, approved: 23 October 2018.

### 2.2. Neuromotor and Behavioral Tests

Animals were assessed for motor coordination via rotarod at weekly intervals from age 5 to 10 weeks. All other behavioral testing was begun at P70.

#### 2.2.1. Rotarod Assay for Motor Coordination (Ataxia)

Mice were tested 4 times daily (two times in the morning and two times in the afternoon) for 2 consecutive days by being placed on a horizontal rod which steadily increases rotational speed from 4 rpm to 40 rpm over 5 min. Temporal latency to fall off the rod was averaged for the 8 trials. 

#### 2.2.2. Open Field Assay for Locomotion

Mice were tested by placement in an open field apparatus (50 × 50 cm) with 40 cm high opaque walls and allowed to explore for 5 min. Animal movement was recorded by an overhead video camera and analyzed by video tracking software (VideoMot 2, TSE Systems, Chesterfield, MO, USA). Average speed was calculated as total distance traveled divided by total time.

#### 2.2.3. Audio Open Field Audiogenic Seizure Test

Mice were tested for audiogenic seizure (AGS) susceptibility as previously described [9]. Using the open field apparatus and video tracking software described above, mice were allowed to explore the open field for 2½ min, then exposed to an 85 dB white noise played continuously through a loud speaker. Average speed during the pre-sound period (pre-sound) and in the initial 10 s period following the onset of white noise (post-sound) was calculated. Trials were stopped if animals seized continuously for more than 15 s.

### 2.3. Histology and Immunohistochemistry

Tissue was collected and processed as previously described [10]. Briefly, mice were deeply anesthetized using 1.2% Avertin and perfused transcardially with 0.1 M sodium phosphate buffer (PB) followed by 4% PFA in PB. Brains were removed, stored in 4% PFA for 24 h, then immersed sequentially in 10%, 20%, and 30% sucrose in PB (24 h per solution). Brains were cut into 40-µm coronal sections. To assay for reactive gliosis, sections were incubated with primary antibody-glial fibrillary acidic protein (GFAP) (Dako #Z0334, Santa Clara, CA, USA). GFAP was visualized using diaminobenzidine tetrahydrochloride (DAB) immunohistochemistry, sections were treated with 0.3% H_2_O_2_, blocked, and incubated in primary antibody overnight at 4 °C. The sections were then probed with the appropriate biotinylated secondary antibody followed by incubation in an avidin–biotin–peroxidase complex (Vectastain Elite ABC Kit, Vector Labs #PK6100, Burlingame, CA, USA) and visualized by peroxidase detection using DAB HRP Peroxidase Substrate Kit (Vector Labs #Sk4100, Burlingame, CA, USA).

### 2.4. Silver Staining and Quantification

Silver staining was performed using the FD Neurosilver Kit II (FD Neurotechnologies #PK301, Columbia, MD, USA) according to the manufacturer’s instructions. The optical density of GFAP immune reactivity and silver staining in the inferior colliculus (IC) and the decussation of the superior cerebellar peduncle (XSCP) was quantified at the same coronal levels, −5.02 and −4.48 mm, respectively. Brightfield images (5× objective) were captured with a digital camera mounted on a Zeiss microscope (Axioskop2, Oberkochen, Germany), imported into ImageJ analysis software (National Institutes of Health, Bethesda, MD, USA), and converted to black-and-white images. Contours were drawn around the area of interest and the adjacent area (as background control). Mean optical densities were determined as differences between area of interest and control.

### 2.5. Protein Extraction and Glutathione Peroxidase (Gpx) Activity Assay

Mice were euthanized by CO_2_ inhalation and brains were quickly removed. Brains were cut in half along the longitudinal fissure, with one hemisphere being snap-frozen in liquid nitrogen for use as a whole-brain sample, and the other dissected into smaller regions and then snap-frozen. Frozen tissues were lysed by sonication in CelLytic MT Cell Lysis Reagent (Sigma-Aldrich #C3228, St. Louis, MO, USA) containing protease inhibitors (Calbiochem #539134, San Diego, CA, USA) and centrifuged at 14,000× *g* for 10 min at 4 °C. Supernatants were collected, and the protein concentrations were measured using a Nanodrop NP-1000 spectrophotometer (Waltham, MA, USA).

Samples were diluted to 4 μg/μL in glutathione peroxidase (Gpx) buffer consisting of 50 mM phosphate buffer + 5 mM ethylenediaminetetraacetic acid, pH = 7.4. Then, 25 μL of sample was loaded into a 96-well plate and incubated at 37 °C for 3 min in 45 μL of master mix: 12.5 μL of 30 mM glutathione (Sigma CAS 70-18-8), 12.5 μL of 30 μg/mL glutathione reductase (Sigma 9001-48-3), and 20 μL of 1 mM NADPH (Sigma 2646-71-1)). Next, 5 μL of 6 mM tert-butyl hydroperoxide solution were added to each sample and the plate was read by a Spectramax M3 plate reader (Molecular Devices) at 340 nm for 10 min. Using Softmax Pro 6.2.1 software (Sunnyvale, CA, USA), the Gpx activity was measured as the reduction rate of tert-butyl hydroperoxide catalyzed by the samples upon the oxidation of glutathione and reduced NADPH. A unit of activity was defined as the consumption of 1 μmol of NADPH per min, calculated from the equation (Vjmax × Vt/Vs)/(0.0062 × D), using 0.0062 μM^−1^ cm^−1^ as the extinction coefficient for NADPH at 340 nm.

### 2.6. Statistical Analysis

Statistical tests and sample numbers varied with each experiment and are indicated in the text and/or figure legends. Data were analyzed and plotted using GraphPad Prism version 5 Software (Softmax). All results are represented as mean ± SEM. Significance was determined by a *p*-value of <0.05.

## 3. Results

### 3.1. Male and Female DKOSe Mice Exhibited Impaired Motor Coordination Prior to 10 Weeks of Age

**Figure 1:**
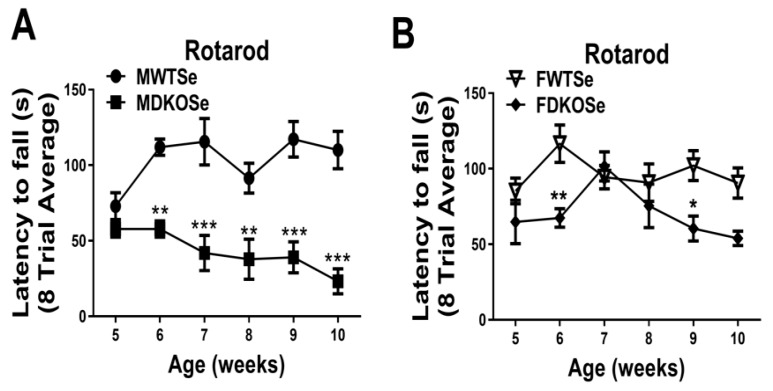
Rotarod performance in wild-type mice supplemented with Se (WTSe) and double knockout mice supplemented with Se (DKOSe). (**A**) Latency to fall of male WTSe and DKOSe mice from 5 to 10 weeks of age. (**B**) Latency to fall of female WTSe and DKOSe mice from 5 to 10 weeks of age. (Rotarod two-way ANOVA: Males interaction F_(5,60)_ = 3.12, *p* = 0.0145, Genotype F_(1,60)_ = 102.44, *p* < 0.0001, week NS; Females interaction NS, Genotype F_(1,60)_ = 19.08, *p* < 0.0001, week NS; *n* = 6 all groups. Bonferroni’s: * *p* < 0.05, ** *p* < 0.01, *** *p* < 0.001). All values reported as Mean ± SEM.

We previously reported that MDKOSe mice performed poorly in the rotarod assay at both 8 and 12 weeks of age [9,10]. In this study, we assessed male and female WTSe and DKOSe mice at weekly intervals from age 5 to 10 wks (P35 to P70). The FDKOSe and MDKOSe mice both exhibited a significant decline in performance on the rotarod starting at 6 wks of age (Figure 1A,B); however, FDKOSe mice exhibited a milder deficit. The FDKOSe mouse “latency to fall” times (amount of time mice can stay on the rotarod) were shorter than FWTSe, but longer than MDKOSe mice. Thus, even with SeH_2_O supplementation, FDKOSe mice developed a neuromotor deficit during adolescence.

### 3.2. Neurological Dysfunction in FDKOSe Mice was Modulated by SeH_2_O Removal

**Figure 2:**
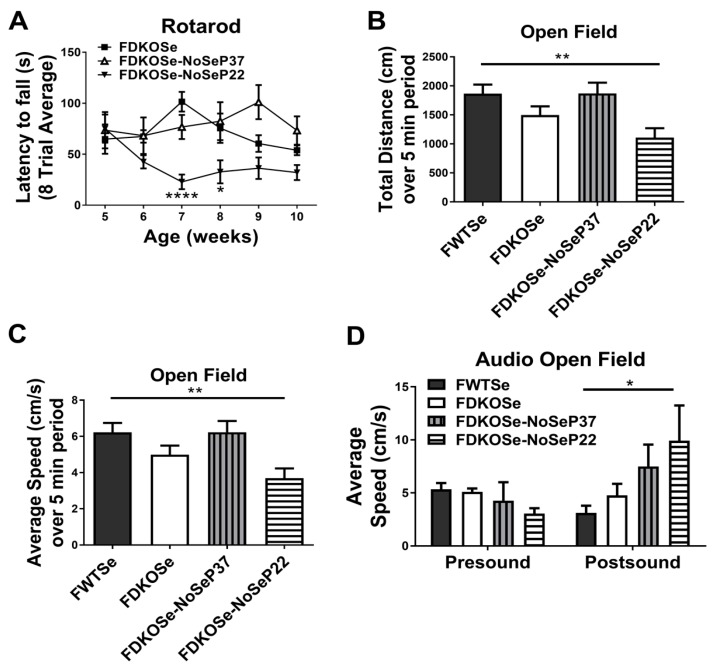
Effect of SeH_2_O withdrawal age on motor function and seizures in female double knockout mice supplemented with Se (FDKOSe). (**A**) Latency to fall during rotarod assay, (**B**) total distance traveled, and (**C**) average speed over 5-min period in the open field test, and (**D**) Average speed 2½ min prior (presound) and 10 s after (postsound) 85 dB white noise played during open field sudiogenic seizure assay. Assays were carried out on female wild-type mice supplemented with Se (FWTSe), FDKOSe, FDKOSe-NoSeP37 (supplemental Se removed at P37), and FDKOSe-NoSeP22 mice at 10 weeks of age. (**A**) Rotarod two-way ANOVA: Females Interaction F_(15,111)_ = 1.968, *p* = 0.0238, Genotype F_(3,111)_ = 25.1, *p* < 0.0001, week NS; FWTSe, FDKOSe, and FDKOSe-NoSeP22, *n* = 6, NoSeP37, *n* = 4; *,**** indicate time-wise significant differences between FDKOSe and FDKOSe-NoSeP22 according to Bonferroni’s multiple comparisons test. There were no significant differences between FDKOSe and FDKOSe-NoSeP37 at any time point. FDKOSe-NoSeP37 values were significantly higher than FDKOSe-NoSeP22 at 7(*), 8(*), 9(**), and 10(*) weeks. (**B**–**D**). Open field one-way ANOVA: Distance *p* = 0.0084, Average Speed, *p* = 0.0084, FWTSe and FDKOSe *n* = 12, P37 *n* = 7, P22 *n* = 10; Audio open field two-way ANOVA: Interaction NS, *n* = 11 all groups except FDKOSe-NoSeP37, *n* = 4; Bonferroni’s multiple comparisons test: * *p* < 0.05, ** *p* < 0.01). All values reported as mean ± SEM.

The MDKO mice required SeH_2_O supplementation to survive past weaning but still developed neurological dysfunction and susceptibility to audiogenic seizures (AGS) starting at puberty [9]. This was not the case with FDKOSe mice on the same SeH_2_O supplementation. Since a Se-deficient diet aggravates the phenotype in both SELENOP KO and Scly KO mice, we removed SeH_2_O supplementation from FDKOSe mice at either post-natal day 22 (NoSeP22) or 37 (NoSeP37), and tested for motor coordination and susceptibility to seizure development. The FDKOSe-NoSeP22 mice performed significantly worse than FDKOSe mice on the rotarod (Figure 2A). These reduced “latency to fall” times were comparable to MDKOSe mice (Supplementary Materials Figure S1A). Surprisingly, removing Se supplementation at P37 had no significant effect, as FDKOSe-NoSeP37 did not perform worse than FDKOSe mice at any time point.

Assessment of locomotion and exploratory behavior in the open field assay showed that FDKOSe-NoSeP22 mice traveled significantly less (Figure 2B) over a 5-min period and at significantly slower speeds (Figure 2C) than FWTSe mice. In the AGS assay, FDKOSe-NoSeP22 mice exhibited increased wild running in response to an 85 dB white noise (Figure 2D). In both the open field test and AGS test, FDKOSe and FDKOSe-NoSeP37 showed similar, yet weaker, trends that were not statistically significant.

### 3.3. FDKOSe-NoSeP22 Mice Exhibited Neuroinflammation and Neurodegeneration in Auditory Brain Nuclei and Motor Tracts

**Figure 3:**
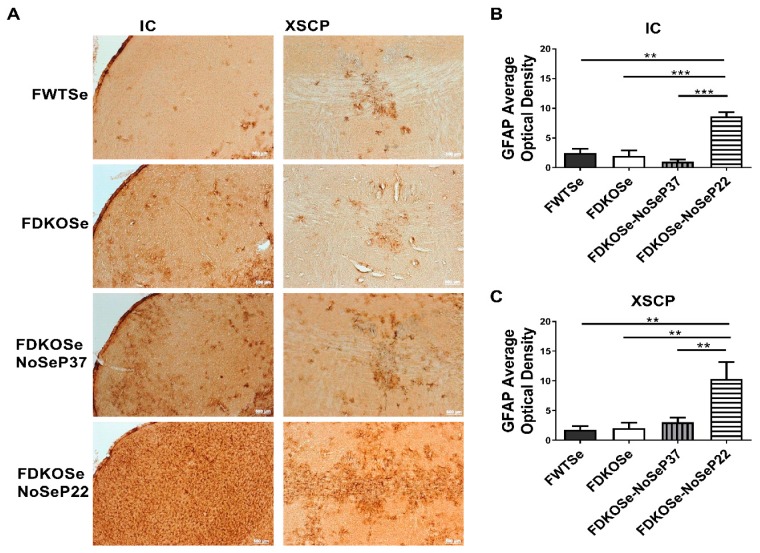
Effect of SeH_2_O (supplemental Se water) withdrawal age on glial fibrillary acidic protein (GFAP) immunoreactivity in female double knockout mice supplemented with Se (FDKOSe). (**A**) Representative images of inferior colliculus (IC) and the decussation of the superior cerebellar peduncle (XSCP) of female wild-type mice supplemented with Se (FWTSe), FDKOSe, FDKOSe-NoSeP37 (supplemental Se removed at P37), and FDKOSe-NoSeP22 GFAP mice at 10 weeks of age. Quantification of GFAP immunoreactivity in (**B**) IC and (**C**) XSCP. (One-way ANOVA IC *p* = 0.0003, XSCP *p* = 0.0151; Bonferroni’s multiple comparisons test: ** *p* < 0.005, *** *p* < 0.0005; *n* = 3 all groups). All values reported as mean ± SEM.

We next assessed neurodegeneration in the same areas of the brain found to be affected in MDKOSe mice, the inferior colliculus (IC) and decussation of the superior cerebellar peduncle (XSCP). The FDKOSe-NoSeP22 mice exhibited significantly higher levels of GFAP average optical density in the IC and XSCP compared to all other groups (Figure 3A–C), implying substantial astrocytic activation in response to neuronal damage, consistent with our prior findings with MDKO mice (15).

**Figure 4:**
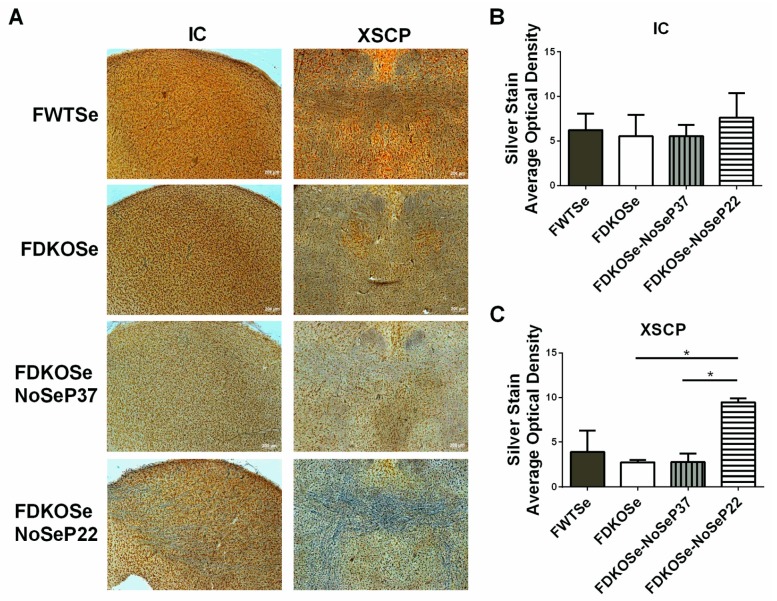
Effect of SeH_2_O (supplemental Se water) withdrawal age on silver staining in female double knockout mice supplemented with Se (FDKOSe). (**A**) Representative images of silver staining in inferior colliculus (IC) and the decussation of the superior cerebellar peduncle (XSCP) of female wild-type mice supplemented with Se (FWTSe), FDKOSe, FDKOSe-NoSeP37 (supplemental Se removed at P37), and FDKOSe-NoSeP22 mice at 10 weeks of age. Quantification of average optical density in (**B**) IC and (**C**) XSCP. (one-way ANOVA IC NS, XSCP *p* = 0.0187; Bonferroni’s multiple comparisons test: * *p* < 0.5; *n* = 3 all groups). All values reported as mean ± SEM.

Although whole brain Gpx activity levels were not significantly different between FDKOSe and FDKOSe-NoSeP22 mice, both had significantly decreased levels compared to FWTSe mice (Supplementary Materials Figure S2). In the brain stem, there was a trend of decreased Gpx activity levels, with FDKOSe-NoSeP22 having the lowest levels. Silver staining, used to detect degenerating neuronal somata, axons, and terminals which become argyrophilic as they deteriorate, showed significant neurodegeneration in the axons of the motor tracts in the XSCP of FDKOSe-NoSeP22 mice (Figure 4A–C).

## 4. Discussion

Previously, we reported that male mice with combined KO of SELENOP and Scly exhibit decreased viability beginning in adolescence, and those that survive develop severe neurodegeneration, impaired motor performance, and audiogenic seizure (AGS) [9]. In subsequent studies, we demonstrated that these impairments are likely due to competition between the brain and testes for Se utilization during puberty and can be attenuated via prepubescent castration [10]. Herein, we report that FDKOSe mice develop a similar, yet milder neurological phenotype than MDKOSe mice. Although FDKOSe mice exhibited motor coordination deficits on the rotarod, the degree of impairment was not as severe as in MDKOSe mice (Figure 1). The FDKOSe mice also did not suffer from AGS, one of the striking features observed in MDKOSe mice. Additionally, no signs of neurodegeneration were observed in FDKOSe mice (Figure 3 and Figure 4). The neurological effects of SELENOP/Scly DKO on females are likely less severe than males, at least in part, due to the absence of the brain–testes competition paradigm.

Interestingly, restriction of dietary Se intake just prior to puberty in FDKOSe mice exacerbated motor impairments and induced neurodegeneration (Figure 2, Figure 3 and Figure 4) not seen in FDKOSe mice with unrestricted Se access. Neuroinflammation and neurodegeneration were evident in the same areas of the brain, IC and XSCP, as previously observed in MDKOSe mice indicating that the same developmental factors and/or mechanistic pathways may be disrupted in both male and female DKO mice. Surprisingly, Se intake restriction at P22 led to AGS susceptibility in adulthood in FDKOSe mice (Figure 2). Thus, challenging FDKOSe mice with Se deficiency during puberty induces a neurological phenotype similar to that seen in MDKOSe mice. Removing supplemental Se during the latter stages of puberty (P37), produced none of the detrimental effects that pre-pubescent removal did.

Although FDKOSe-NoSeP22 mice were challenged with SeH_2_O removal for a longer time period than their -NoSeP37 counterparts prior to testing, and it could, thus, be argued that time post-SeH_2_O removal is a determining factor in motor performance, it is important to take note of several qualities of the rotarod data (Figure 2A): (1) The performance of FDKOSe-NoSeP22 mice became significantly poor at 7 weeks of age, corresponding to 4 total weeks of SeH_2_O removal, and remained at the same level of performance thereafter. (2) FDKOSe-NoSeP37 mice at 9 weeks of age, corresponding to 4 weeks of SeH_2_O removal within this group, showed no deficits and still performed significantly better than their -NoSeP22 counterparts. This holds true for subsequent timepoints when comparing both “SeH_2_O removal” groups either according to age or time post-SeH_2_O removal (Supplementary Materials Figure S1B). (3) While FDKOSe-NoSeP22 mice displayed neuromotor deficits fairly early on, FDKOSe-NoSeP37 performance was never worse than the original testing (4 weeks) for the group and never statistically different from FDKOSe performance at any timepoint. Altogether, these findings further emphasize puberty as a period in central nervous system development during which Se utilization is critical in mice. While males are more susceptible to neurological disturbances, females develop similar symptoms when Se availability is more severely compromised.

AGS development in MDKOSe could result from GABAergic system disturbances including decreased GAD_67_ and parvalbumin (PV)^+^ interneuron density in the IC, which we previously observed [9]. Indeed, mice with genetic deletion of GAD_65_ have a lower seizure threshold [11]. To determine whether FDKOSe mice develop neuromotor deficits via mechanisms similarly to MDKOSe, studies on PV^+^ interneurons and GABAergic system defects in the IC, XSCP, and related areas during puberty are needed.

## 5. Conclusions

The data presented in this study demonstrate that FDKOSe mice suffer from neurological deficits that, although initially not as severe as in MDKOSe mice, become intensified if SeH_2_O is removed throughout puberty, leading to poor motor function and AGS development. Therefore, Se availability during this critical time is necessary for the prevention of neurological impairment in male and female mice. Overall, this study improves our understanding of sex differences in Se utilization. Further investigation is needed to elucidate the mechanistic factors underlying the sex-specific differences observed when Se metabolism is perturbed.

## Supplementary Materials

The following are available online at https://www.mdpi.com/2076-3921/8/4/110/s1, Figure S1. Comparison of female double knockout mice supplemented with Se until P22 (FDKOSe)-NoSEP22rotarod performance to male double knockout mice supplemented with Se (MDKOSe) and FDKOSe-NoSeP37 mice. Figure S2. Effect of SeH_2_O (supplemental Se water) withdrawal on glutathione peroxidase (Gpx) activity in female double knockout mice supplemented with Se (FDKOSe).
