# Supplementary material for: Disruption of Selenium Handling During Puberty Causes Sex-Specific Neurological Impairments in Mice
Source: Antioxidants (Basel). 2019 Apr 24;8(4):110. doi: 10.3390/antiox8040110 (PMC6523490; doi:10.3390/antiox8040110)
Supplement: Supplementary file 1 [file antioxidants-08-00110-s001.pdf]

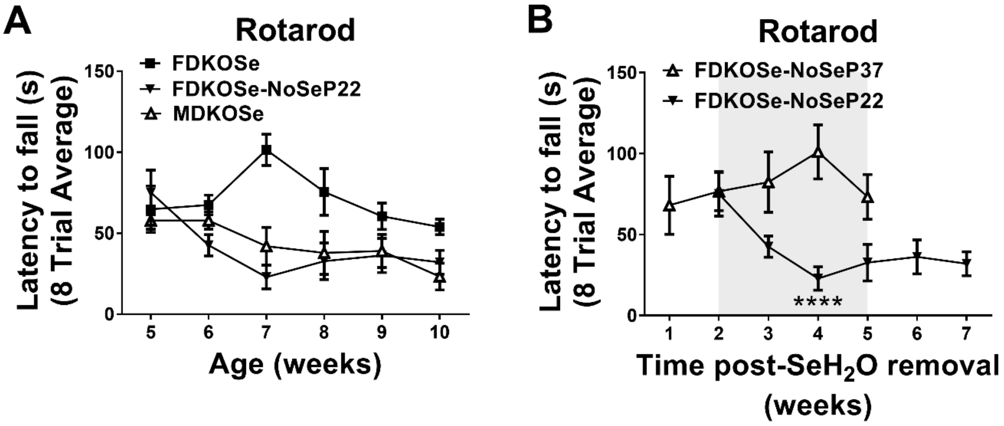

Figure S1. Comparison of female double knockout mice supplemented with Se until P22 (FDKOSe)-NoSEP22rotarod performance to male double knockout mice supplemented with Se (MDKOSe) and FDKOSe-NoSeP37 mice. (**A**) SeH_2O (supplemental Se water) removal from FDKOSe mice reduces rotarod performance to a level comparable to MDKOSe mice. (**B**) Comparison of FDKOSe mice with SeH_2O removed at P22 and P37 according to the number of weeks post-SeH_2O removal (x-axis). Grey shading shows timepoints at which data was available for both groups. These data were compared via two-way ANOVA: Interaction $F_{(3,35)} = 2.97$ $p < 0.05$, Time of SeH_2O removal $F_{(1,35)} = 1.085$ $p < 0.0001$, Weeks NS; FDKOSe-NoSeP37 $n = 4$, and FDKOSe-NoSeP22 $n = 6$. Bonferroni's multiple comparisons test: ****$p < 0.0001$ at 4 weeks post SeH_2O removal. C57/BL6 mice aged P70 were used. All values reported as Mean ± SEM.

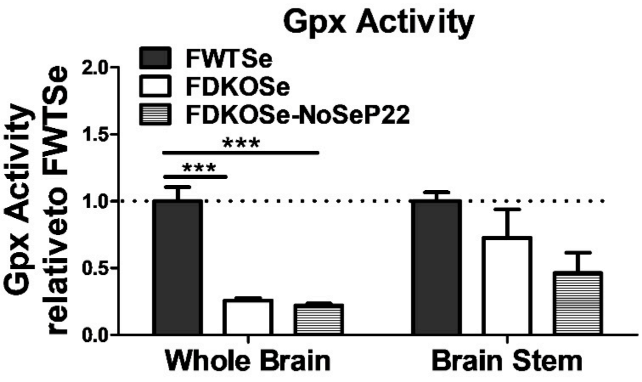

Figure S2. Effect of SeH_2O (supplemental Se water) withdrawal on glutathione peroxidase (Gpx) activity in female double knockout mice supplemented with Se (FDKOSe). Gpx activity in whole brain and brain stem relative to female wild-type mice supplemented with Se (FWTSe) values. One-way ANOVA: Whole Brain $p < 0.0001$; Brain Stem NS $p = 0.0820$; Bonferroni's multiple comparisons test: ***$p < 0.0005$, $n = 3$ to 4 in triplicates. Female C57/BL6 mice aged P70 were used. Data presented as mean ± SEM.
